# Acute ghrelin changes food preference from a high‐fat diet to chow during binge‐like eating in rodents

**DOI:** 10.1111/jne.12463

**Published:** 2017-04-02

**Authors:** T. Bake, K. T. Hellgren, S. L. Dickson

**Affiliations:** ^1^ Department of Physiology/Endocrine Institute of Neuroscience and Physiology The Sahlgrenska Academy at the University of Gothenburg Gothenburg Sweden

**Keywords:** binge eating, dietary preference, food choice, ghrelin, GHS‐R1A, high‐fat diet

## Abstract

Ghrelin, an orexigenic hormone released from the empty stomach, provides a gut–brain signal that promotes many appetitive behaviours, including anticipatory and goal‐directed behaviours for palatable treats high in sugar and/or fat. In the present study, we aimed to determine whether ghrelin is able to influence and/or may even have a role in binge‐like eating behaviour in rodents. Accordingly, we used a palatable scheduled feeding (PSF) paradigm in which *ad lib*. chow‐fed rodents are trained to ‘binge’ on a high‐fat diet (HFD) offered each day for a limited period of 2 hours. After 2 weeks of habituation to this paradigm, on the test day and immediately prior to the 2‐hour PSF, rats were administered ghrelin or vehicle solution by the i.c.v. route. Remarkably and unexpectedly, during the palatable scheduled feed, when rats normally only binge on the HFD, those injected with i.c.v. ghrelin started to eat more chow and chow intake remained above baseline for the rest of the 24‐hour day. We identify the ventral tegmental area (VTA) (a key brain area involved in food reward) as a substrate involved because these effects could be reproduced, in part, by intra‐VTA delivery of ghrelin. Fasting, which increases endogenous ghrelin, immediately prior to a palatable schedule feed also increased chow intake during/after the schedule feed but, in contrast to ghrelin injection, did not reduce HFD intake. Chronic continuous central ghrelin infusion over several weeks enhanced binge‐like behaviour in palatable schedule fed rats. Over a 4‐week period, GHS‐R1A‐KO mice were able to adapt and maintain large meals of HFD in a manner similar to wild‐type mice, suggesting that ghrelin signalling may not have a critical role in the acquisition or maintenance in this kind of feeding behaviour. In conclusion, ghrelin appears to act as a modulating factor for binge‐like eating behaviour by shifting food preference towards a more nutritious choice (from HFD to chow), with these effects being somewhat divergent from fasting.

## 1 Introduction

The determining factors and mechanisms controlling dietary food choice behaviour remain some of the most important and yet less chartered landscapes in obesity research. This may be because, in contrast to food intake, which is under tight physiological control and involves prominently unconscious intrinsic homeostatic mechanisms, food choice is more vulnerable to a host of additional determining factors that include, for example, cognitive, societal, familial, environmental and socio‐economic factors. From an evolutionary perspective, food choice is important for survival, ensuring that, in times of famine, animals would seek out, select and even feast on energy‐dense foods as they become available.

In rodents, it is possible to steer macronutrient choice towards fat by an overnight fast,^1^ although little is known about the metabolic signals involved. Recently, we hypothesised that the stomach‐derived hormone, ghrelin, could provide such a signal.^2^ Ghrelin is released in association with hunger^3^ and acts within the brain to bring about a feeding response,^4^, ^5^ engaging both homeostatic pathways in the hypothalamus,^6^ as well as reward pathways important for food anticipatory^7^, ^8^ and food‐motivated behaviour.^9^, ^10^, ^11^, ^12^ Indeed, we found that ghrelin can redirect food choice but not as expected.^2^ In these studies, rats were offered a free *ad lib*. choice of normal chow, lard (animal fat) and sucrose pellets and, at baseline, were consuming large amounts of lard. As is the case for fasting, acute ghrelin injection to the brain ventricles or to the ventral tegmental area (VTA; a key reward node) increased the intake of fat. However, remarkably, under the influence of ghrelin, there was a three‐fold increase in the amount of regular chow consumed in these high fat‐consuming rats.

In the present study, we sought to explore the effects of ghrelin on food choice in rats and mice trained to show binge‐like behaviour for a high‐fat diet (HFD). We reasoned that it would be difficult to change food choice during the high fat binge. “Binge eating” is a term used to describe excessive consumption of large amounts of mostly energy‐dense food during a short period of time. In humans, it is marked by some level of emotional distress, such as loss of control, disgust, guilt, depression and embarrassment. Binge eating disorder (BED) is the clinical manifestation of binge eating, and results in obesity and individuals becoming overweight.^13^ The consummatory aspects of this behaviour can be induced in rodents using a schedule feeding paradigm in which regular chow diet is supplemented by a palatable food (eg HFD) that is offered for a restricted period each day. When exposed to this palatable schedule feeding paradigm, rats and mice can eat up to 63% and 86%, respectively, of their entire daily caloric intake from the palatable food.^14^ The term “binge‐like eating” is used to describe this entrainable feeding behaviour.

The aim of the present study was to determine whether ghrelin impacts on binge‐like behaviour for HFD, offered as a 2‐hour daily schedule feed as an optional supplement to *ad lib*. chow.^14^, ^15^, ^16^ We were especially interested to determine whether ghrelin could steer dietary choice towards chow in this binge model in which the rats are highly motivated to consume large amounts of the HFD. Given that bingeing is a complex behaviour that promotes unhealthy food consumption beyond metabolic needs, we investigated whether the effects of ghrelin on binge‐like behaviour could be driven from a key reward area, the VTA, which is a known target for ghrelin to direct goal‐directed behaviour for palatable foods.^9^, ^10^, ^11^, ^12^ We also sought a role for endogenous ghrelin signalling in these effects by performing schedule feeding studies in mice that lack the ghrelin receptor, GHS‐R. Finally, because ghrelin is considered to operate as a circulating hunger hormone, we aimed to determine the impact of fasting (that increases endogenous ghrelin levels) on food preference during and after scheduled feeding.

## 2 Materials and Methods

### 2.1 Animals

Four different animal experimental studies were performed. Three of the studies were undertaken in male Sprague‐Dawley rats (Charles River, Germany). Immediately upon arrival at the animal facility at 7 weeks of age and a body weight of 200‐220 g, the rats were housed in a room under reversed 12 : 12 hour light/dark cycle (lights on depending on the study design) and allowed to acclimatise for at least 1 week in groups prior to the experimental procedures.

The fourth study was done in male GHS‐R knockout (KO) mice and their wild‐type (WT) littermates that were bred in‐house from a colony kept at Experimental Biomedicine at the University of Gothenburg.^9^ The mice were generated from crosses between heterozygous breeding pairs. After weaning at 3 weeks of age, they were housed in group cages with their littermates. The mice were kept under a 12 : 12 hours light‐dark cycle with lights on at 06.00 h. Once they reached 7 weeks of age, male mice were single housed and transferred to a reversed light/dark cycle (lights on 16.00 hours) and acclimatised for 2 weeks prior to the experimental procedures.

All animals had *ad lib*. access to standard maintenance chow (#2016; 22% protein, 66% carbohydrate, 12% fat by energy, 3.00 kcal/g; Harlan Labs, Indianapolis, IN, USA) and water unless otherwise specified. They were kept in standardised nonbarrier conditions at a temperature in the approximate range 20‐22°C and a humidity of approximately 50%. The studies were carried out with ethical permission obtained from the local animal ethics committee at the University of Gothenburg. Ethical permit numbers were 45‐2014 (rats), 156‐12 (mice) and 155‐12 (breeding of genetically modified mice).

### 2.2 Dietary manipulation and food intake analysis

For dietary manipulation a palatable HFD (#D12492; 20% protein, 20% carbohydrate, 60% fat by energy, 5.24 kcal/g; Research Diets, New Brunswick, NJ, USA) was used in both rat and mouse studies. Arguably, the HFD diet can be considered “unhealthier” than the chow diet because it contains much more fat and also less fibre (6.5% by weight for HFD and 15.2% by weight for chow diet). The carbohydrate part of the HFD contained mainly maltodextrin and sucrose (12.3% and 6.8% by energy). During the palatable schedule feeding paradigm (PSF‐paradigm), the animals were given access to HFD for a limited time of 2 hours beginning in the middle of the dark phase (at 6 hours after lights off). The timing was chosen to replicate the feeding paradigm described by Berner et al.^17^ and Bake et al.^14^, ^15^, ^16^ However, unlike in these previous studies, HFD was always offered in addition to chow in order to obtain information about the role of ghrelin on food preference during the 2‐hour palatable schedule feed (2 hr‐PSF).

After surgery, all rats were housed in an automated feeding and drinking monitoring system (TSE LabMaster; TSE Systems, Bad Homburg, Germany) that measured food consumption by weight in two separate food sensors. The PSF‐paradigm commenced after 1 week of acclimatisation to the cages and was conducted manually for at least 2 weeks prior to the start of injection. Data were manually analysed for each rat for HFD and chow intake at 1, 2, 4, 6, 18 and 24 hours after injection.

The mice were housed in standard cages. Food was given manually and food intake was measured by weighing the food given and the food left prior and after the 2 hr‐PSF. Chow was measured at the same time intervals. Food intake was measured by weight (g) and then converted to energy (kcal). In all studies, body weights were recorded at frequent intervals (eg either three times a week or prior and 24 hours after injection).

### 2.3 Study 1: Impact of i.c.v. ghrelin injection or fasting on PSF in rats

For study 1, rats (n=16) were implanted with an i.c.v. guide cannula into the lateral ventricle under anaesthesia induced by i.p. injection of a Ketaminol (75 mg/kg; Intervet, Boxmeer, the Netherlands) and Rompun (10 mg/kg; Bayer, Leverkusen, Germany) mixture. Rats were positioned in a stereotaxic frame (Model 942; David Kopf Instruments, Tujunga, CA, USA). The skull bone was exposed and the skull sutures were identified. Bregma was located and used as origin for coordinates. Holes for guide cannulae and anchoring screws (#MCS1x2; Agnthos, Lidingö, Sweden) were drilled. A 26 gauge cannula was positioned according to coordinates (0.9 mm posterior to bregma, ±1.6 mm lateral to the midline and 2.5 mm ventral of the skull surface) and fixed in place with anchoring screws and dental cement (#7508, #7509; Agnthos). A dummy cannula (#C313DC; Bilaney, Sevenoaks, UK) was inserted into the guide cannula to prevent obstruction. After surgery, the rats received an analgesic (Rimadyl; Orion Pharma Animal Health, Sollentuna, Sweden) and were single housed and allowed to recover for 1 week. Intracerebroventricular cannula placement and the projection length of the injector (2.0 or 2.5 mm) was confirmed in conscious rats with a 2 μL angiotensin II (10 ng/μL; #1158; Tocris, Bristol, UK;) injection. Placement was considered correct if the rat drank water within 5 minutes and more than 5 mL within 30 minutes following the injection. The rats were then habituated to the PSF‐paradigm for 2 weeks to display binge‐like feeding behaviour for HFD. Injections of ghrelin (1 μg or 2 μg in 1 μL; #1463; Tocris) or artificial cerebrospinal fluid (aCSF; #3525; Tocris) were performed in a cross‐over design. These doses had previously been shown to induce a feeding response in rats.^4^ Injections were performed just before start of the 2 hr‐PSF (at 14.00 h; lights on 20.00 h) and a minimum of 48 hours in between injections. Food consumption was analysed at a total of six time points after injection (1, 2, 4, 6, 18 and 24 hours). To allow comparison with natural hunger, at the end of the ghrelin vs vehicle injection study, the same rats were fasted for 16 hours prior to schedule feeding start and food intake was analysed at the same time points.

### 2.4 Study 2: Impact of intra‐VTA ghrelin injection on PSF in rats

The study protocol used for study 2 was the same as in study 1 with the exception that the VTA was targeted in rats (n=15). The VTA is a brain area important for food reward and ghrelin is able to regulate food intake and food motivated behaviour at the level of the VTA.^10^, ^18^ The coordinates for VTA unilateral cannula placement were: 5.7 mm posterior to bregma, ±0.75 mm lateral to the midline and 6.5 mm ventral of the skull surface with a projection of 2 mm. VTA cannula placement was verified with a post mortem of 0.5 μL of India ink. Rats with an incorrect placement were excluded from the analysis. Injections of ghrelin (0.5 μg or 1 μg in 0.5 μL; Tocris) or aCSF were performed in a cross‐over design. These doses had previously been shown to increase feeding in rats.^2^, ^18^ Injections were performed over 1 min (flow rate of 0.5 μL/min). Lights on was at 17.00 h.

### 2.5 Study 3: Impact of chronic i.c.v. ghrelin administration on PSF in rats

The rats (n=16) were implanted with primed osmotic minipumps (ALZET #2004; Agnthos; infusion over 28 days, flow rate of 0.25 μL/h) that were connected via vinyl tubing to a cannula into the lateral ventricle (ALZET brain infusion kit #2; Agnthos; same coordinates as in Study 1). Cannula placement was verified with a post mortem injection of 2.0 μL of India ink into the cannula after the tubing was disconnected. All rats had the correct placement. Rats were divided by body weight into two groups, with eight rats receiving ghrelin and eight rats receiving aCSF as control. Delivery started immediately after minipump implantation. Ghrelin was delivered in aCSF at a flow rate of 0.5 μg/h, which is a dose that has previously been shown to increase food intake and body weight.^11^, ^19^ Rats were fed for 10 days on standard chow after minipump implantation to confirm the chronic effect of ghrelin on food intake and body weight under the control condition. Afterwards, all rats were for fed for 18 days on the PSF‐paradigm with HFD as described above. Lights on was at 17.00 h.

### 2.6 Study 4: PSF in GHS‐R KO mice

In a fourth study, using genetically modified mice that lack the ghrelin receptor (GHS‐R KO), we further investigated the role for endogenous ghrelin signalling to initiate and maintain binge‐like behaviour. We exposed the mice to the same PSF‐paradigm used for the rats at 9 weeks of age (ie 1 week after single housing). The mice were allowed to acclimatise to single housing and the reversed light cycle for 2 weeks (lights on 16.00 h) and were then divided into four groups. Group 1 consisted of GHS‐R KO mice that had 2 hours of access to HFD beginning in the middle of the dark phase (at 6 hours after lights off) in addition to chow (KO‐PSF, n=7), as did group 2 that consisted of WT mice (WT‐PSF, n=6). Groups 3 (KO‐con, n=6) and 4 (WT‐con, n=6) were used as control groups and only had access to *ad lib*. chow. The food intake was, however, measured at the same time (at 6 and 8 hours after lights off) to control for the disturbance that was caused to the mice in groups 1 and 2 and to be able to compare their feeding behaviour. The PSF‐paradigm was undertaken over 4 weeks. The statistical analysis of the food intake data was performed for week 4 only. The body composition of the mice was performed at the end of week 4 and analysed by dual‐energy X‐ray absorptiometry.

### 2.7 Statistical analysis

All statistical analysis was conducted using spss, version 22 (IBM, Armonk, NY, USA). In the acute delivery studies (Studies 1 and 2), data were checked for normal distribution and heterogeneity and then analysed by one‐way analysis of variance (ANOVA) followed by Tukey's post‐hoc tests. Cumulative HFD and chow data were analysed separately and also combined as total intake at several time points after injection. In Study 3, data were checked for normal distribution and heterogeneity and then analysed by an independent samples *t*‐tests on each measurement day after minipump implantation. In Study 4, data were checked for normal distribution and heterogeneity and then analysed by two‐way ANOVA for the factors of genotype (WT vs KO) and feeding regime (scheduled feeding vs control feeding) and also for interaction between these factors. Post‐hoc and planned comparison were assessed by Tukey's test. All data are presented as the mean±SEM. *P*<.05 was was considered statistically significant for all data.

## 3 Results

### 3.1 Acclimatisation to the PSF paradigm

Rats took less than a week to adapt to the PSF‐paradigm. Intake of HFD increased rapidly over 4 days (see Supporting information, Fig. S1A) and chow intake during the 2 hr‐PSF decreased to almost 0 on day 2 (see Supporting information, Fig. S1B). Chow intake during the remaining 22 hours decreased more slowly over several days (see Supporting information, Fig. S1C). Total caloric intake reached a maximum after 4 days (see Supporting information, Fig. S1D). After 2 weeks of training the PSF‐paradigm, the rats were consuming 62.1% of their total daily energy intake from the HFD (see Supporting information, Fig. S1F), offered for only 2 hours/day. During the 2 hr‐PSF, HFD was the only food consumed (99.2% preference) (see Supporting information, Fig. S1E).

### 3.2 Study 1: Intracerebroventricular ghrelin or fasting: food intake and food choice in rats during and after exposure to a PSF paradigm

**Figure 1:**
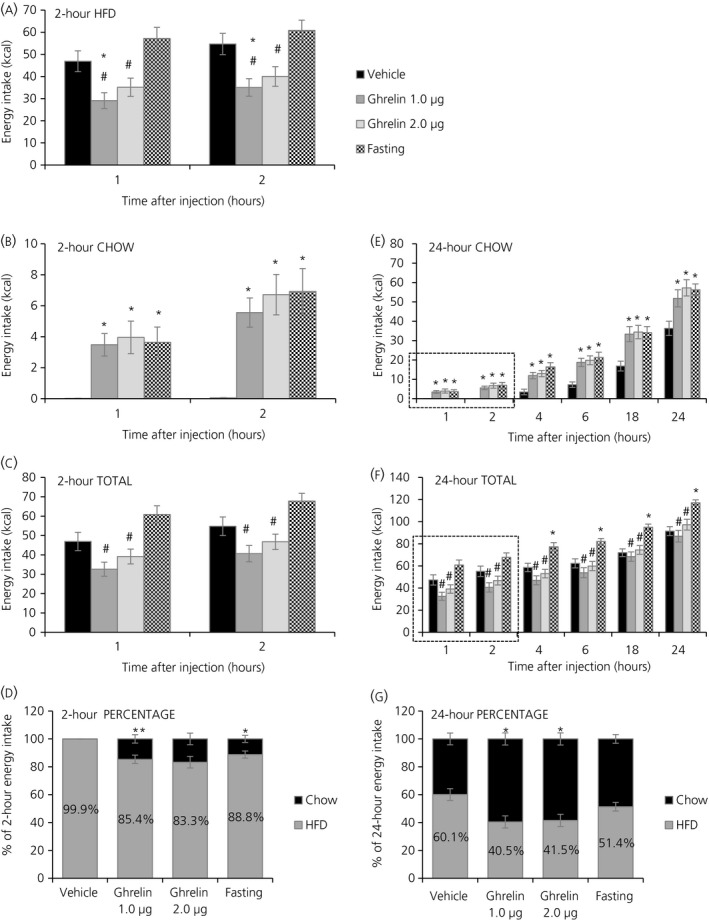
Effects of acute i.c.v. ghrelin injection and a 16 hours fast on energy intake and food preference in rats exposed to a palatable schedule feeding (PSF)‐paradigm. For injection studies, artificial cerebrospinal fluid (aCSF) was administered as vehicle control solution and ghrelin was administered at two different doses (1 and 2 μg). (A) Cumulative energy intake from high‐fat diet (HDF) during a 2 hr‐palatable schedule feed (2 hr‐PSF). (B) Cumulative energy intake from chow during the 2 hr‐PSF. (C) Cumulative total energy intake (combined from HFD and chow) during the 2 hr‐PSF. (D) Percentage of HFD in relation to chow during the 2 hr‐PSF. (E) Cumulative energy intake from chow up to 24 hours post‐injection. (F) Cumulative total energy intake (combined from HFD and chow) up to 24 hours post‐injection. (G) Percentage of HFD in relation to chow in the 24 hours post‐injection period. Data are presented as the mean±SEM. *P<.05 vs vehicle, ^#^ *P*<.05 vs fasting by one‐way ANOVA (n=16 rats)

After 2 weeks of PSF‐paradigm, vehicle‐injected rats were consuming 60.1% of their total daily energy intake from the HFD (Figure 1G) and HFD was the only food consumed during the 2 hr‐PSF (99.9% preference) (Figure 1D). When ghrelin was acutely injected into the lateral ventricle, there was a decrease in cumulative HFD intake (relative to vehicle‐injected controls) at 1 hour and at 2 hours post‐injection with the lower ghrelin dose (one‐way ANOVA: *P*<.001 at 1 and 2 hours; Tukey's post‐hoc test: *P*=.026 at 1 hour and *P*=.014 at 2 hours) (Figure 1A). Both ghrelin doses also gave a decrease in HFD intake during the 2 hr‐PSF compared to fasting (Tukey's post‐hoc test: ghrelin 1 μg, *P*<.001 at 1 and 2 hours; ghrelin 2 μg, *P=*.004 at 1 hour and *P*=.010 at 2 hours) (Figure 1A). At the same time that HFD decreased, chow intake increased at 1 and 2 hours post‐injection with both ghrelin doses (one‐way ANOVA: *P*<.001 at 1 and 2 hours; Tukey's post‐hoc test: ghrelin 1 μg, *P*=.016 at 1 hour, *P*=.003 at 2 hours; ghrelin 2 μg, *P=*.005 at 1 hour and *P*>.001 at 2 hours) ( Figure 1B). Fasting also increased chow intake during and after the 2 hr‐PSF (Tukey's post‐hoc test: *P*=.013 at 1 hour, *P*>.001 at 2 hours) (Figure 1B). Total energy intake (from HFD and chow combined), however, was unchanged at the same time points with ghrelin injections compared to vehicle but decreased compared to fasting (one‐way ANOVA: *P*<.001 at 1 and 2 hours; Tukey's post‐hoc test: ghrelin 1 μg, *P*<.001 at 1 and 2 hours; ghrelin 2 μg, *P*=.00 at 1 hour and *P*=.006 at 2 hours) (Figure 1C). The percentage of HFD intake in relation to chow changed towards lower HFD with both ghrelin doses and with fasting during the 2 hr‐PSF (one‐way ANOVA: *P*<.001; Tukey's post‐hoc test: ghrelin 1 μg, *P*=.002; ghrelin 2 μg, *P*<.001; Fasting, *P*=.026) (Figure 1D).

The ghrelin effect with both doses and the fasting effect persisted for the observed 24 hours post‐injection for both chow (one‐way ANOVA: *P*<.001 at 4, 6, 18 and 24 hours; Tukey's post‐hoc test: ghrelin 1 μg vs vehicle, *P*=.003 at 4 and 6 hours, *P*=.007 at 18 hours, *P*=.025 at 24 hours; ghrelin 2 μg vs vehicle, *P*=.001 at 4 hours and at 6 hours, *P*=.004 at 18 hours, *P*=.001 at 24 hours; fasting vs vehicle, *P*<.001 at 4 and 6 hours, *P*=.005 at 18 hours, *P*=.003 at 24 hours) (Figure 1E) and total energy intake (one‐way ANOVA: *P*<.001 at 4, 6, 18 and 24 hours; Tukey's post‐hoc test: fasting vs vehicle, *P*=.006 at 4 hours, *P*=.004 at 6 hours, *P*>.001 at 18 and 24 hours; fasting vs ghrelin 1 μg, *P*<.001 at 4, 6, 18 and 24 hours; fasting vs ghrelin 2 μg, *P*<.001 at 4, 6 and 18 hours; *P*=.013 at 24 hours) (Figure 1F). The percentage of HFD ingested in relation to 24 hours chow changed towards lower HFD with both ghrelin doses but not with fasting (one‐way ANOVA: *P*<.003; Tukey's post‐hoc test: ghrelin 1 μg, *P*=.006; ghrelin 2 μg, *P*=.011) (Figure 1G).

### 3.3 Study 2: Intra‐VTA ghrelin: food intake and food choice in rats during and after exposure to a PSF paradigm

**Figure 2:**
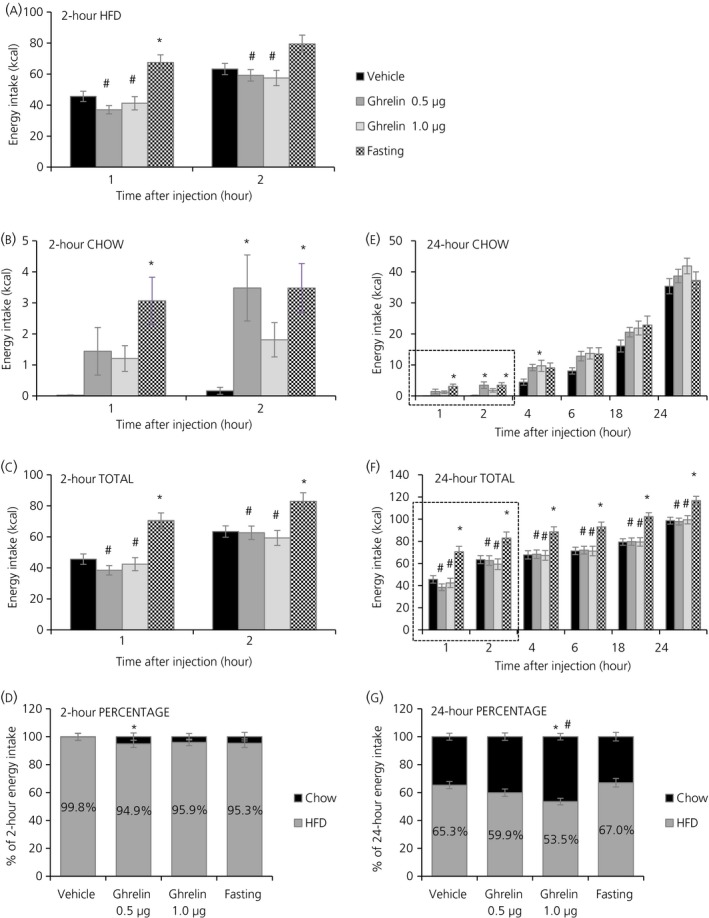
Effects of acute intra‐VTA ghrelin and a 16 hours fast on energy intake and food preference in rats exposed to a palatable schedule feeding (PSF)‐paradigm. Artificial cerebrospinal fluid (aCSF) was administered as vehicle control solution and ghrelin was administered at two different doses (0.5 and 1 μg). (A) Cumulative energy intake from a high‐fat diet (HDF) during a 2‐hour palatable schedule feed (2 hr‐PSF). (B) Cumulative energy intake from chow during the 2 hr‐PSF. (C) Cumulative total energy intake (combined from HFD and chow) during the 2 hr‐PSF. (D) Percentage of HFD in relation to chow during the 2 hr‐PSF. (E) Cumulative energy intake from chow up to 24 hours post‐injection. (F) Cumulative total energy intake (combined from HFD and chow) up to 24 hours post‐injection. (G) Percentage of HFD in relation to chow in the 24 hours post‐injection period. Data are presented as the mean±SEM. *P<.05 vs vehicle, ^#^ *P*<.05 vs fasting by one‐way ANOVA (n=15 rats)

After 2 weeks of exposure to the PSF‐paradigm, vehicle‐injected rats were consuming 65.3% of their total daily energy intake from HFD (Figure 2G) and HFD was the only food consumed during the schedule feed (99.8% preference) (Figure 2D). Intra‐VTA injection of ghrelin gave a similar but less pronounced feeding response compared to i.c.v. injections. During the 2 hr‐PSF, there was a decrease in cumulative HFD intake at 1 and 2 hours post‐injection with both ghrelin doses vs fasting (one‐way ANOVA: *P*<.001 at 1 hour and *P*=.009 at 2 hours; Tukey's post‐hoc test: ghrelin 0.5 μg, *P*<.001 at 1 hour, *P*=.025 at 2 hours; ghrelin 1 μg, *P*<.001 at 1 hour and *P*=.010 at 2 hours) (Figure 2A) and fasting increased HFD intake vs vehicle at 1 hour (Tukey's post‐hoc test: *P*=.002 at 1 hour) (Figure 2A). At the same time as HFD decreased, chow intake increased at 2 hours post‐injection with the lower ghrelin doses (one‐way ANOVA: *P*=.007 at 1 hour and *P*=.005 at 2 hours; Tukey's post‐hoc test: ghrelin 0.5 μg, *P*=.011 at 2 hours) (Figure 2B). Fasting also increased chow intake at 1 and 2 hours (Tukey's post‐hoc test: *P*=.003 at 1 hour, *P*=.013 at 2 hours) (Figure 2B). Total energy intake (from HFD and chow combined) was unchanged at the same time points with ghrelin injections compared to vehicle but fasting increased the total energy intake (one‐way ANOVA: *P*<.001 at 1 hour and *P*=.005 at 2 hours; Tukey's post‐hoc test: fasting vs vehicle, *P*<.001 at 1 hour, *P*=.029 at 2 hours; fasting vs ghrelin 0.5 μg, *P*<.001 at 1 hour, *P*=.028 at 2 hours; fasting vs ghrelin 1 μg, *P*<.001 at 1 hour, *P*=.006 at 2 hours) (Figure 2C). The percentage of HFD intake in relation to chow changed towards lower HFD with the lower ghrelin doses during the 2 hr‐PSF (one‐way ANOVA: *P*=.020; Tukey's post‐hoc test: ghrelin 0.5 μg vs vehicle, *P*=.026) (Figure 2D).

The ghrelin effect with both doses persisted for the observed 24 hours post‐injection on chow (one‐way ANOVA: *P*=.038; Tukey's post‐hoc test: ghrelin 1 μg vs vehicle, *P*=.048) (Figure 2E) and total energy intake, which was increased with fasting (one‐way ANOVA: *P*=.003 at 4 hours, *P*<.001 at 6 and 18 hours, *P*=.002 at 24 hours; Tukey's post‐hoc test: fasting vs vehicle, *P*=.007 at 4 hours, *P*=.002 at 6 hours, *P*<.001 at 18 hours, *P*=.005 at 24 hours; fasting vs ghrelin 0.5 μg, *P*=.013 at 4 hours, *P*=.005 at 6 hours, *P*<.001 at 18 hours, *P*=.004 at 24 hours; fasting vs ghrelin 1 μg, *P*=.006 at 4 hours, *P*=.002 at 6 hours, *P*<.001 at 18 hours, *P*=.008 at 24 hours) (Figure 2F). The percentage of HFD intake in relation to 24 hours chow changed towards lower HFD with the higher ghrelin doses but not with fasting (one‐way ANOVA: *P*=.007; Tukey's post‐hoc test: ghrelin 1 μg vs vehicle, *P*=.025; ghrelin 1 μg vs fasting, *P*=.009) (Figure 2G).

### 3.4 Study 3: Food intake and food choice in PSF rats receiving chronic i.c.v. delivery of ghrelin

**Figure 3:**
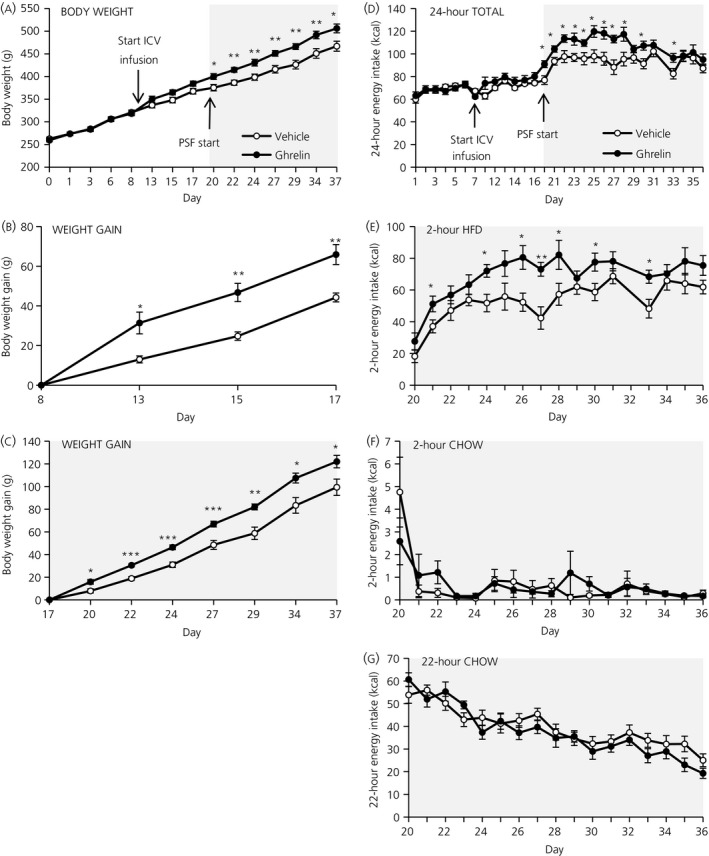
Effects of chronic i.c.v. ghrelin delivery over 4 weeks in rats exposed to a palatable feeding schedule (PSF)‐paradigm. (A) Body weight development over pre‐surgery, chow feeding and scheduled feeding phases. (B) Body weight gain during 10 days of chow feeding. (C) Body weight gain over 18 days exposure to the PSF‐paradigm. (D) Total daily energy intake over pre‐surgery, chow feeding and palatable scheduled feeding phases. (E‐G) Energy intake during palatable scheduled feeding phase: (E) Energy intake from a high‐fat diet (HDF) during the 2‐hour palatable schedule feed (2 hr‐PSF); (F) Energy intake from chow during the 2 hr‐PSF; and (G) Energy intake from chow during the remaining 22 hours. Ghrelin (closed circles) vs vehicle (open circles). Data are presented as the mean±SEM. *P<.05; **P<.01; ***P<.001 by independent samples *t*‐test (n=8 rats per group)

When ghrelin was delivered chronically into the lateral ventricle, body weight increased from the first day of the schedule feeding phase in the ghrelin vs the vehicle group (day 20, *P*=.021; day 22, *P*=.003; day 24, *P*=.007; day 27, *P*=.004; day 29, *P*=.004; day 34, *P*=.009; day 37, *P*=.019; by an independent samples *t*‐test), but not during the preceding chow feeding phase (Figure 3A). Body weight gain, calculated from the last day of the respective preceding phase, increased in both the chow feeding phase (day 13, *P*=.012; day 15, *P*=.002; day 17, *P*=.003; by an independent samples t‐test) (Figure 3B) and in the scheduled feeding phase (day 20, *P*=.011; day 22, *P*<.001; day 24, *P*<.001; day 27, *P*=.002; day 29, *P*=.002; day 34, *P*=.010; day 37, *P*=.025; by an independent samples *t*‐test) (Figure 3C) in ghrelin vs vehicle treatment.

Chronic ghrelin delivery did not increase the total daily energy intake when the rats were fed for 10 days on standard chow only. When exposed to the PSF‐paradigm for 18 days, energy intake increased for a limited time comprising 9 consecutive days (day 20, *P*=.012; day 21, *P*=.027; day 22, *P*=.023; day 23, *P*=.023; day 24, *P*=.046; day 25, *P*=.013; day 26, *P*=.011; day 27, *P*=.004; day 28, *P*=.023; day 30, *P*=.018; day 33, *P*=.044; by an independent samples *t*‐test) in the ghrelin group but then decreased to intake levels of the vehicle group (Figure 3D). The increased total daily energy intake in the ghrelin group is the result of an increase of HFD during the 2 hr‐PSF on several days (day 21, *P*=.045; day 24, *P*=.010; day 26, *P*=.010; day 27, *P*=.002; day 28, *P*=.048; day 30, *P*=.030; day 33; *P*=.014; by an independent samples *t*‐test) (Figure 3E). However, chow intake during the 2 hr‐PSF was not changed by chronic ghrelin delivery (Figure 3F) and chow intake during the remaining 22 hours also stayed unchanged (Figure 3G).

### 3.5 Study 4: Food intake and food choice in PSF GHS‐R KO mice

**Figure 4:**
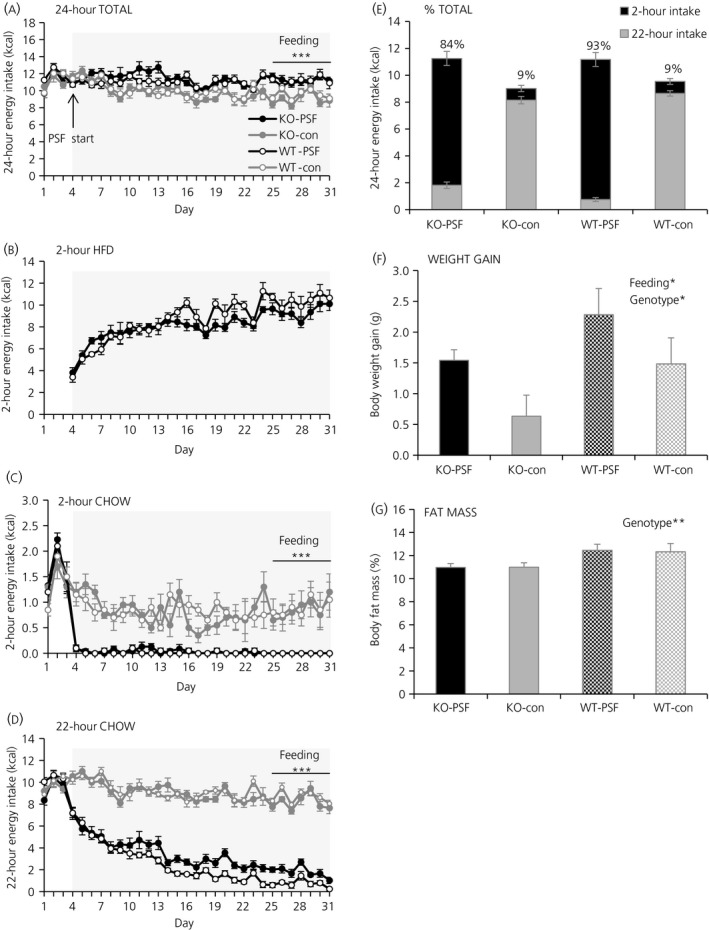
Palatable schedule feeding in ghrelin receptor knockout mice. Over 4 weeks, GHS‐R1A knockout (KO; closed circles) mice and their wild‐type (WT; open circles) littermates were either fed normal chow ad libitum (WT‐PSF and WT‐con; grey) or exposed to a palatable feeding schedule (PSF)‐paradigm (KO‐PSF and WT‐PSF; black). (A) Total daily energy intake. (B) Energy intake from a high‐fat diet (HDF) during the 2‐hour palatable scheduled feed (2 hr‐PSF). (C) Energy intake from chow during the 2 hr‐PSF. (D) Energy intake from chow during the remaining 22 hours. (E) Percentage of energy intake during the 2 hr‐PSF and during the remaining 22 hours. (F) Body weight gain. (G) Body fat mass as percentage of body weight gain after 4 weeks on the respective feeding paradigms. Data are presented as the mean±SEM. *P<.05; **P<.01; ***P<.001 by two‐way ANOVA with factors feeding paradigm (PSF‐paradigm vs con) or genotype (KO vs WT) (n=6‐7 mice per group)

Over 4 weeks, GHS‐R KO mice and their WT littermates were fed either normal chow or were exposed to the PSF‐paradigm. For statistical analysis, the mean intake values of week 4 of schedule feeding were used. Energy intake from HFD during the 2 hr‐PSF was similar between the two groups exposed to the PSF‐paradigm (KO‐PSF vs WT‐PSF) (Figure 4A). The amount of HFD consumed was 84% of total daily calories in KO‐PSF mice and 93% in WT‐PSF mice (Figure 4B). Chow intake during the 2 hr‐PSF (Figure 4C), chow intake during the remaining 22 hours (Figure 4D) and total daily energy intake over 24 hours (Figure 4E) did not differ between the genotypes (KO vs WT), although they were significantly affected by the dietary paradigm (2 hours intake, *P*<.001, PSF<con; 22 hours intake, *P*<.001, PSF<con; 24 hours intake, *P*<.001, PSF>con; two‐way ANOVA).

Body weight gain after 4 week of schedule feeding was significantly affected by both genotypes (*P*=.031, KO<WT, two‐way ANOVA) and feeding paradigm (*P*=.022, PSF>con, two‐way ANOVA) (Figure 4F), whereas the body fat mass was only significantly affected by genotype (*P*=.010, KO<WT, two‐way‐ANOVA) (Figure 4G).

## 4 Discussion

Palatable schedule feeding, in which rodents are offered a palatable treat for a limited time each day as a supplement to their regular chow, evokes a powerful, binge‐like behavioural response. Rodents learn to expect a regular daily treat and will consume a large proportion of their daily calories from it.^20^, ^21^ In two different studies reported here, *ad lib*. chow‐fed rats were given access to a HFD for 2 hours each day, during which time, in the control state, they consumed only HFD, comprising 60% and 65.3% of their total daily energy intake. Notably, total 24‐hour food intake is much greater in rats with limited (2 hours) access to a palatable food than in those with *ad lib*. access to this food.^17^ This suggests that limiting access to a palatable food may increase its reward value and hence increase its consumption, a behaviour which is hedonically driven. In the present study, we demonstrate that some aspects of this scheduled feeding binge‐like behaviour for HFD can be altered by brain delivery of ghrelin.

Previous studies have shown that ghrelin levels are increased prior to access to a palatable food (chocolate) offered for a limited time each day in a schedule feeding paradigm, and that ghrelin is important for the expression of anticipatory hyperlocomotor activity for the palatable food.^8^ In the present study, we explored how ghrelin could alter food choice during the schedule feed and also total daily energy consumption, both of which are important for obesity development. Given that ghrelin is orexigenic^4^, ^5^ and increases motivated behaviour for sugar^9^, ^10^, ^11^ and fat,^12^ we expected to discover that ghrelin would increase HFD consumption during the 2‐hour limited access period and have an overall orexigenic effect. Notably and unexpectedly, when ghrelin was administered by acute i.c.v. injection immediately prior to the limited 2 hr‐PSF, the rats started to eat more regular chow, and continued to do so during the rest of the 24‐hour day. During the 2 hr‐PSF, i.c.v. ghrelin resulted in an overall reduction in total kcal eaten, and hence a shift in dietary choice, because less HFD was consumed. Indeed, the proportion of 24‐hour energy intake from HFD (which was 60% in the controls receiving i.c.v. vehicle solution) was much lower (~40%) after i.c.v. ghrelin delivery. Thus, the central ghrelin signalling system appears to redirect food selection towards chow (a more nutritious option, with less fat and more fibre) in rats trained to binge on a HFD (present study), as well as in rats (as previously reported) consuming a large proportion of their daily intake from fat in an *ad lib*. free choice situation.^2^ Although we do not yet have an explanation for this change in dietary choice by acute ghrelin, we may speculate that ghrelin may increase preference for a “healthier” diet (with more fibre and less fat) or that this effect is somehow linked to the effects of ghrelin with respect to altering substrate utilisation (less fat burning).^22^

In humans, and especially in certain clinical groups, intermittent calorie restriction or dieting is associated with binge eating behaviour.^23^, ^24^ This has been modelled in animals: food restriction has been shown to enhance binge‐like eating in rats exposed to a PSF‐paradigm.^25^, ^26^ In the present study, we explored the impact of a 16‐hour fast on food choice during the 2 hr‐PSF and also on total daily energy intake. Given that ghrelin levels are increased by fasting^27^, ^28^ we expected to find some similarities in binge‐like behaviour in rats fasted for 16 hours and those administered ghrelin i.c.v. It might be expected that these hungry rats would binge on the energy‐dense HFD because previous studies have shown that preference for fat increases after an overnight fast.^1^ However, we found that, during the 2 hr‐PSF, 16‐hour fasted rats started to eat regular chow at a level similar to that induced by i.c.v. ghrelin. However, unlike i.c.v. ghrelin, fasting drove an overall orexigenic response because total 24‐hour energy intake was increased, without a compensatory decrease in HFD consumption during the 2 hr‐PSF. The fact that fasting does not further increase HFD (or indeed total energy intake) during a 2 hr‐PSF, could reflect the fact that the rats have eaten as much as is physically possible during this initial period after the fast. Therefore, it was interesting to monitor food intake over the entire 24‐hour day, for which it was very clear that chow intake and total energy intake were increased by i.c.v. ghrelin relative to vehicle controls, although there was no significant change in 24‐hour food choice. Collectively, these data suggest that the total amount of food consumed during/after a binge can be enhanced by fasting but not by ghrelin. Our data do, however, point to a role for ghrelin during fasting with respect to promoting the consumption of chow, even in hungry rats highly motivated to consume HFD.

The VTA‐NAcc pathway appears to be recruited by ghrelin for controlling food‐motivated behaviour but not spontaneous food intake.^18^ VTA delivery of ghrelin has been shown to enhance fasting induced hyperphagia.^29^ The data presented here support the hypothesis that the VTA could contribute to the effects of ghrelin with respect to enhancing chow intake and altering food choice in rats exposed to a PSF‐paradigm. When ghrelin was delivered unilaterally into the VTA, we were able to reproduce, albeit to a lesser extent, some of the effects of i.c.v. ghrelin delivery. At least during the second hour of the palatable schedule feeding, chow intake was increased and, as was the case for i.c.v. ghrelin, intra‐VTA ghrelin did not cause an orexigenic effect during or after the palatable schedule feed but did alter 24‐hour dietary choice (at least at the higher dose).

Next, we aimed to determine whether animals with altered ghrelin signalling behave differently when exposed to a palatable feeding schedule. *Ad lib*. chow fed ghrelin receptor KO mice and their WT littermates were placed on a PSF‐paradigm. We did not detect any difference between genotypes for any of the feeding parameters measured during or after the 2 hr‐PSF and body weight gain did not diverge. This would suggest that ghrelin signalling is not required for the acquisition or expression of binge‐like behaviour in mice. We should not be surprised by the lack of a “binge” phenotype in the ghrelin receptor‐KO mice because they also appear normal in other aspects of energy balance including food intake and adiposity on a standard diet.^30^ Such studies typically attribute the lack of phenotype in the ghrelin receptor KO mice to compensatory processes during developmental and/or to redundancy in the pathways involved, which could also be the case for binge‐like behaviour.

In the chronic i.c.v. ghrelin infusion study, we followed the acquisition of palatable schedule feeding behaviour. For the first 2 weeks of ghrelin delivery, the rats were only fed normal chow, during which time the body weights started to diverge. When they were exposed to the PSF‐paradigm (again, 2 hours of HFD access in addition to *ad lib*. chow), the body weight digressed even further in the ghrelin‐infused rats as a result of an overall increase in the total food consumed. Unlike acute i.c.v. ghrelin treatment, chronic i.c.v. ghrelin delivery had no impact on regular chow intake (during or after the daily 2 hr‐PSF) but did increase HFD consumption during the schedule feed. We do not know why chronic i.c.v. ghrelin differs from acute i.c.v. ghrelin for its effects on dietary choice in the PSF‐paradigm. In the chronic situation, i.c.v. ghrelin is highly orexigenic, amplifying the overall amount of calories consumed in 24 hours. It may be that chronic i.c.v. ghrelin mimics a chronic hyperghrelinaemic state and that the brain interprets this as one of energy deficit, favouring the consumption of energy dense food when it becomes available (ie during the schedule feeding periods in our model). Arguably, however, chronic i.c.v. ghrelin administration does not represent the normal physiological situation in which dynamic changes in ghrelin levels around mealtimes may be important, for example, to avoid down‐regulation or desensitisation of the receptor.^31^ We can view ghrelin as an acute modulator of food intake, sending a hunger signal that promotes food intake, organises feeding into meals, and redirecting food choice to include chow as well as fat. However, if ghrelin levels remain high, mimicking an enhanced hunger signal, it may be that increased energy intake is favoured.

In summary, our data provide evidence for a neurobiological action for the hunger hormone, ghrelin, steering dietary choice towards chow, even in rats highly motivated to consume large amounts of HFD in a palatable schedule feeding paradigm. Ghrelin may be able to enhance binge‐like behaviour, although we did not find any evidence indicating that the ghrelin signalling system is required for mice to acquire this behaviour.

## Conflict of interests

All authors declare that they have no conflicts of interest.

## Authors’ Contributions

TB, SLD designed the research study; TB, KTH performed the research; TB, KTH analysed the data; TB, SLD wrote the manuscript.
