# Supplementary material for: Acute ghrelin changes food preference from a high‐fat diet to chow during binge‐like eating in rodents
Source: J Neuroendocrinol. 2017 Apr 2;29(4):10.1111/jne.12463. doi: 10.1111/jne.12463 (PMC5434925; doi:10.1111/jne.12463)
Supplement: Supplementary file 1 [file JNE-29-0-s001.pptx]

## Slide 1
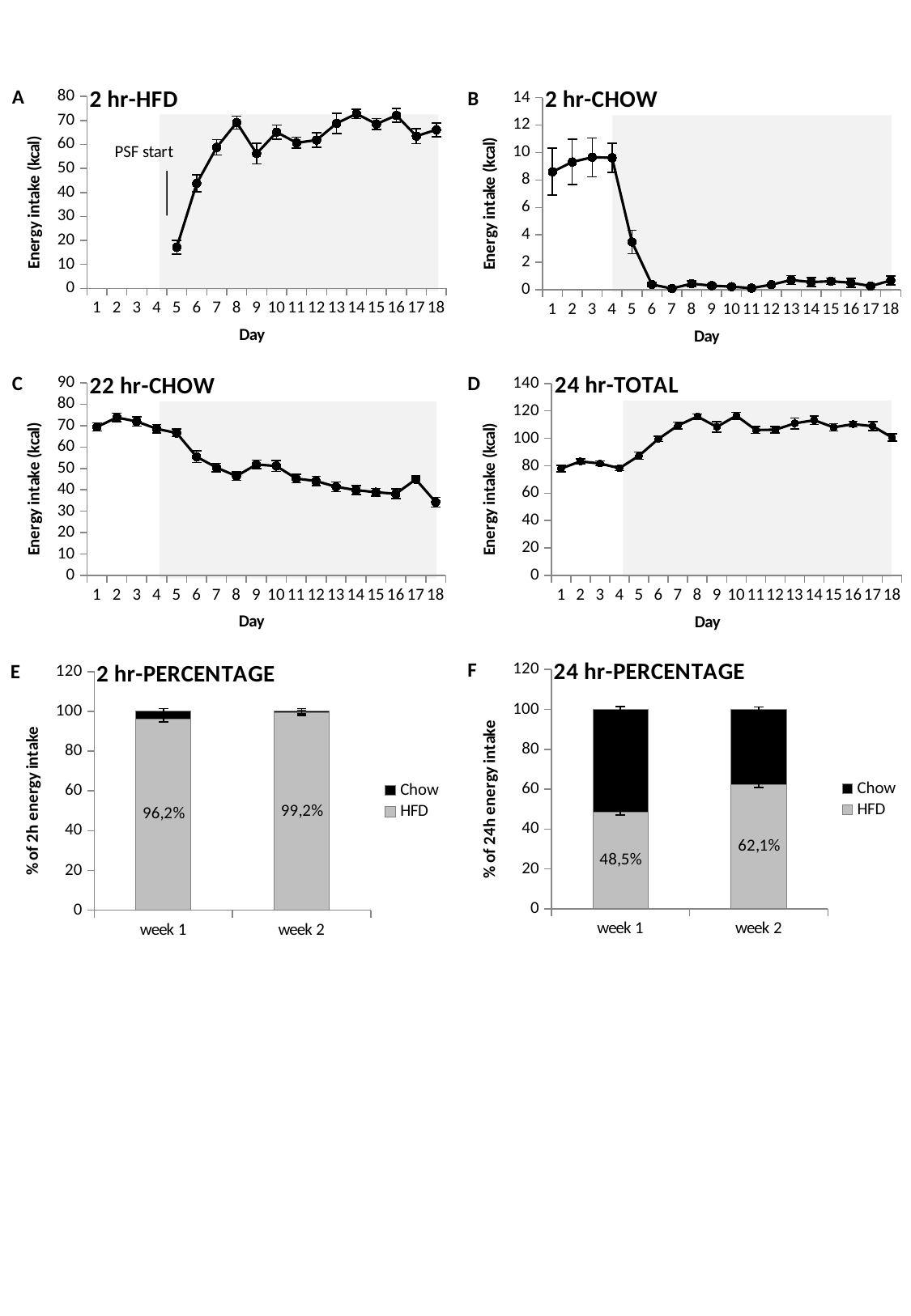

### Chart
| Category | |
|---|---|
| 1 | None |
| 2 | None |
| 3 | None |
| 4 | None |
| 5 | 17.164275000000004 |
| 6 | 43.734350000000006 |
| 7 | 58.78297500000001 |
| 8 | 69.099225 |
| 9 | 56.176075 |
| 10 | 65.090625 |
| 11 | 60.656274999999994 |
| 12 | 61.789425 |
| 13 | 68.76517499999999 |
| 14 | 72.754125 |
| 15 | 68.48025 |
| 16 | 72.1024 |
| 17 | 63.440025 |
| 18 | 66.08295 |
### Chart
| Category | |
|---|---|
### Chart
| Category | |
|---|---|
### Chart
| Category | |
|---|---|
### Chart
| Category | HFD | Chow |
|---|---|---|
| week 1 | 48.46587662389098 | 51.53412337610903 |
| week 2 | 62.057838121406675 | 37.94216187859332 |
### Chart
| Category | HFD | Chow |
|---|---|---|
| week 1 | 96.22935209146554 | 3.770647908534446 |
| week 2 | 99.21283046282817 | 0.7871695371718349 |
